# Supplementary material for: Understanding the role of pyruvate dehydrogenase in Listeria monocytogenes virulence
Source: Infect Immun. Author manuscript; Available in PMC 2026 Jul 17. (PMC13367045; doi:10.1128/iai.00505-25)
Supplement: Supplemental Material [file NIHMS2185584-supplement-Supplemental_Material.docx]

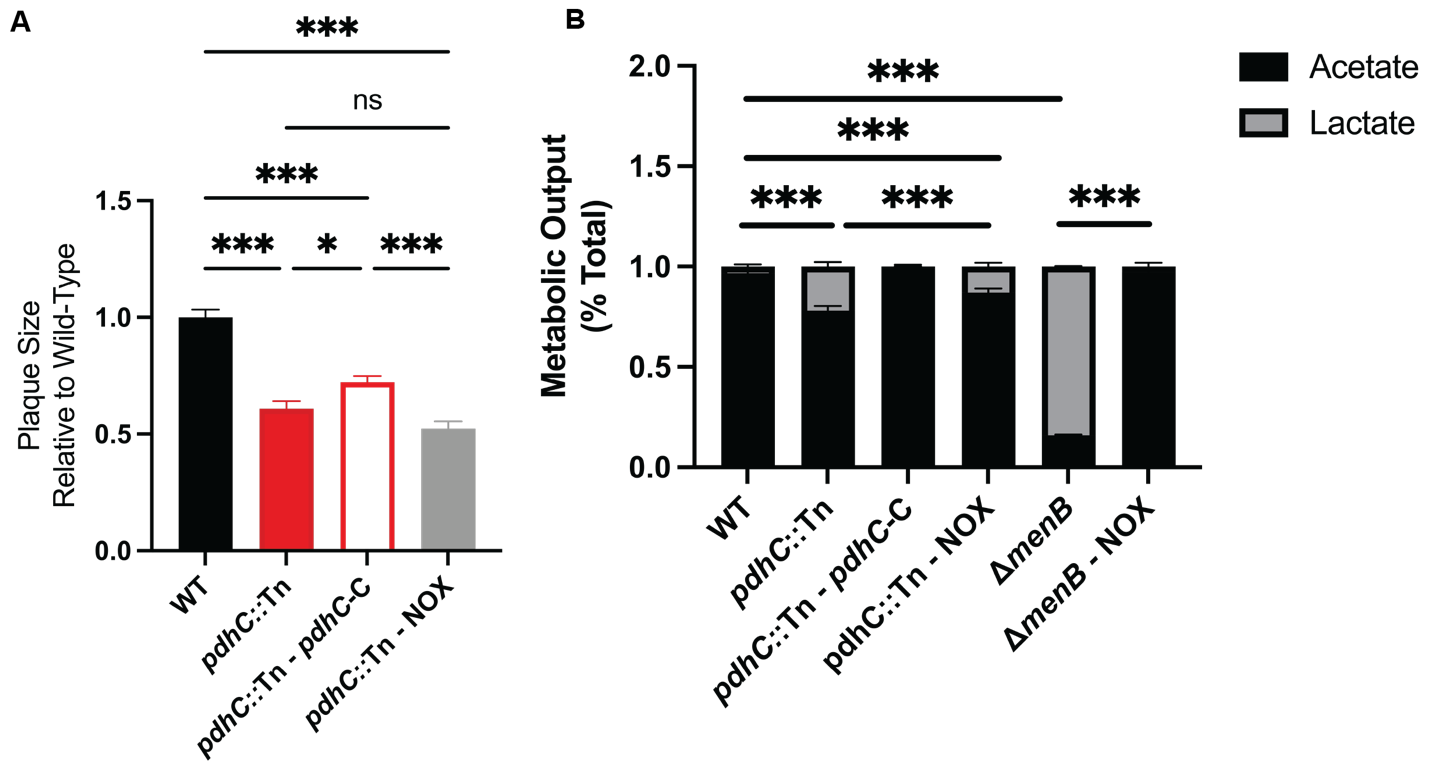


**Supplemental Figure 1. Overexpression of NADH Oxidase, NOX, fails to rescue pdhC::Tn virulence as measured by plaquing assay and fermentative byproducts.** (A) L2 fibroblasts were infected with indicated L. monocytogenes strains at an MOI of 0.5 and were examined for plaque formation 4 days post infection. Assays were performed in biological triplicate and data displayed is the Mean and SEM of a strain’s plaque size relative to WT in one of three representative biological replicates. (B) Note, data for WT, *pdhC::*Tn, and *pdhC*::Tn-*pdhC-*C are reproduced from Figure 2 for comparison. High-performance liquid chromatography (HPLC) was used to quantify fermentation products (acetate and lactate) produced and secreted by the indicated L. monocytogenes strains grown aerobically in BHI medium at 37°C to stationary phase. The mean percentage of acetate and lactate production by each strain was compared to that of the wild-type L. monocytogenes.

| **Strain** | **Description** | **LMRG #** | **LMO #** | **Reference** |
| --- | --- | --- | --- | --- |
| XL1-Blue | competent *E. coli* strain | N/A | N/A | (1) |
| S17 | *E. coli* strain for conjugations into *L. monocytogenes;* Sp^R^ | N/A | N/A | (2) |
| 10403S [JDS 1] | Background *L. monocytogenes* 10403s strain | N/A | N/A | (3) |
| JDS 875 | *pdhA*::Tn | LMRG_00514 | Lmo1052 | This Work |
| JDS 1203 | *pdhC*::Tn | LMRG_00516 | Lmo1054 | (4) |
| JDS 876 | *pdhD*::Tn | LMRG_00517 | Lmo1055 | This Work |
| JDS 1064 | *pdhC*::Tn – *pdhC*-C | N/A | N/A | This Work |
| MJF 363 | *pdhC*::Tn - NOX | N/A | N/A | This Work |
| MJF 368 | *pdhC*::Tn Supp 1 | N/A | N/A | This Work |
| MJF 370 | *pdhC*::Tn Supp 2 | N/A | N/A | This Work |
| MJF 372 | *pdhC*::Tn Supp 3 | N/A | N/A | This Work |
| MJF 374 | *pdhC*::Tn Supp 4 | N/A | N/A | This Work |
| MJF 376 | *pdhC*::Tn Supp 5 | N/A | N/A | This Work |

### **Supplemental Table 1. Bacterial strains used in this study.**





**Supplemental Figure 2. Metabolomic profiling of *pdhC*::Tn-Rex-51Arg-STOP and WT L. monocytogenes glycolytic, fermentative byproduct, and TCA cycle metabolites.** Indicated *L. monocytogenes* strains were grown to mid-log phase (OD₆₀₀ ≈ 0.4) in Listeria Synthetic Medium (LSM) supplemented with 110 mM fructose. Intracellular metabolites were extracted and analyzed via HPLC-MS. Tricarboxylic acid (TCA) cycle intermediates, upper glycolytic metabolites, and lactate were identified based on accurate mass-to-charge (m/z) ratios and retention times using reference values from the KEGG Compound Database, as implemented in MAVEN software. Colored bars represent *pdhC*::Tn-Rex-51Arg-STOP while black bars represent wild-type (WT). Peak areas were quantified and normalized to wild-type levels for each metabolite. Data represent three biological replicates. Statistical comparisons were performed using unpaired two-tailed Student’s t-tests for each metabolite within a strain.

Works Cited:

1. Chen GY, Kao CY, Smith HB, Rust DP, Powers ZM, Li AY, et al. Mutation of the Transcriptional Regulator YtoI Rescues Listeria monocytogenes Mutants Deficient in the Essential Shared Metabolite 1,4-Dihydroxy-2-Naphthoate (DHNA). Freitag NE, editor. Infect Immun. 2019 Dec 17;88(1):e00366-19. doi:10.1128/IAI.00366-19

2. Lauer P, Chow MYN, Loessner MJ, Portnoy DA, Calendar R. Construction, Characterization, and Use of Two Listeria monocytogenes Site-Speciﬁc Phage Integration Vectors. J BACTERIOL. 2002;184.

3. Bécavin C, Bouchier C, Lechat P, Archambaud C, Creno S, Gouin E, et al. Comparison of Widely Used Listeria monocytogenes Strains EGD, 10403S, and EGD-e Highlights Genomic Differences Underlying Variations in Pathogenicity. Casadevall A, editor. mBio. 2014 May;5(2):e00969-14. doi:10.1128/mBio.00969-14

4. Chen GY, McDougal CE, D’Antonio MA, Portman JL, Sauer JD. A Genetic Screen Reveals that Synthesis of 1,4-Dihydroxy-2-Naphthoate (DHNA), but Not Full-Length Menaquinone, Is Required for *Listeria monocytogenes* Cytosolic Survival. Swanson MS, editor. mBio. 2017 May 3;8(2). doi:10.1128/mBio.00119-17
